# Assessment of Suitable Reference Genes for qRT-PCR Normalization in *Eocanthecona furcellata* (Wolff)

**DOI:** 10.3390/insects13090773

**Published:** 2022-08-26

**Authors:** Ying-Na Pan, Ru-Na Zhao, Di Fu, Chun Yu, Chun-Ni Pan, Wei Zhou, Wen-Long Chen

**Affiliations:** Guizhou Provincial Key Laboratory for Agricultural Pest Management of Mountainous Regions, Institute of Entomology, Guizhou University, Guiyang 550025, China

**Keywords:** *Eocanthecona furcellata* (Wolff), reference gene, qRT-PCR, expression stability

## Abstract

**Simple Summary:**

*Eocanthecona furcellata* (Wolff) is an important polyphagous predatory natural enemy insect for agriculture and forestry production. In this paper, we screened nine commonly used reference genes *β-1-TUB*, *RPL4*, *RPL32*, *RPS17*, *RPS25*, *SDHA*, *GAPDH2*, *EF2*, and *UBQ*. Five methods, Ct value, geNorm, NormFinder, BestKeeper, and RefFinder, were used to assess the stability of gene expression at different developmental stages, in different tissues of male and female adults, under different temperatures and starvation treatments. Finally, stable reference genes were screened under different experimental conditions, which laid the foundation for further study of *E. furcellata* gene function.

**Abstract:**

Quantitative reverse transcription–polymerase chain reaction (qRT–PCR) is a widely used tool for measuring gene expression; however, its accuracy relies on normalizing the data to one or more stable reference genes. *Eocanthecona furcellata* (Wolff) is a polyphagous predatory natural enemy insect that preferentially feeds on more than 40 types of agricultural and forestry pests, such as those belonging to the orders Lepidoptera, Coleoptera, and Hymenoptera. However, to our knowledge, the selection of stable reference genes has not been reported in detail thus far. In this study, nine *E. furcellata* candidate reference genes (*β-1-TUB*, *RPL4*, *RPL32*, *RPS17*, *RPS25*, *SDHA*, *GAPDH2*, *EF2*, and *UBQ*) were selected based on transcriptome sequencing results. The expression of these genes in various samples was examined at different developmental stages, in the tissues of male and female adults, and after temperature and starvation treatments. Five algorithms were used, including ΔCt, geNorm, NormFinder, BestKeeper, and RefFinder, to evaluate reference gene expression stability. The results revealed that the most stable reference genes were *RPL32* and *RPS25* at different developmental stages; *RPS17*, *RPL4*, and *EF2* for female adult tissue samples; *RPS17* and *RPL32* for male adult tissue samples; *RPS17* and *RPL32* for various temperature treatments of nymphs; *RPS17* and *RPS25* for nymph samples under starvation stress; and *RPS17* and *RPL32* for all samples. Overall, we obtained a stable expression of reference genes under different conditions in *E. furcellata*, which provides a basis for future molecular studies on this organism.

## 1. Introduction

*Eocanthecona furcellata* (Wolff) is an excellent natural enemy of pests, with predatory capabilities shared by both nymphs and adults [1]. Its abilities are large and diverse, and it can feed on more than 40 types of agriculture and forestry pests, especially those of the order Lepidoptera [2,3,4]. *E. furcellata* may be readily reared in the laboratory and can form large populations. Thus, it is an important natural enemy insect used for biological control [5]. Currently, several studies on *E. furcellata* have focused on its biological characteristics, predatory function, and artificial feeding; however, molecular studies are lacking. In recent years, with the advent of high-throughput sequencing, the transcriptome of *E. furcellata* has been established, which provides a basis for studying its biology and physiology at the molecular level in terms of characterization and function.

Quantitative reverse transcription-polymerase chain reaction (qRT-PCR) is widely used in molecular biology to measure gene expression. It is a rapid and efficient technique with high simplicity and reproducibility, and has the advantages of high sensitivity toward a low concentration of target genes and a high specificity for target genes [6,7]. However, the accuracy of qRT-PCR is influenced by various factors, such as RNA extraction, cDNA synthesis, PCR amplification efficiency, and the stability of the reference or housekeeping genes [8]. Therefore, one or more relatively stable housekeeping genes are required as reference when using qRT-PCR to measure gene expression. Ideal reference genes may be stably expressed in various tissues and cells and under diverse experimental conditions, yet such ideal genes may not exist [9]. With the available molecular techniques, we can find the corresponding stably expressed reference genes for specific conditions. At present, however, there are no relevant studies on the expression stability of reference genes for *E.* *furcellata*.

Therefore, this study aimed to evaluate and select reference genes that are stably expressed under different conditions in *E. furcellata*. To avoid the effects of co-regulation, this study utilized transcriptome sequencing to identify tubulin beta-1 (*β-1-TUB*), 60S ribosomal protein L32 (*RPL32*), 60S ribosomal protein L4 (*RPL4*), succinate dehydrogenase (*SDHA*), 40S ribosomal protein S17 (*RPS17*), 40S ribosomal protein S25 (*RPS25*), glyceraldehyde-3-phosphate dehydrogenase 2 (*GAPDH2*), elongation factor 2 (*EF2*), and ubiquitin (*UBQ*) 9 as housekeeping genes, which were selected from different functional classes and gene families. The expression stability of these candidate genes was also analyzed at different developmental stages, in the tissues of male and female adults, and under different temperature and starvation time treatments using five algorithms, including ΔCt, geNorm version 3.5 (https://genorm.cmgg.be/), NormFinder version 0.953 (http://moma.dk/normfinder-software), BestKeeper version 1.0 (http://www.gene-quantification.de/bestkeeper.html), and RefFinder (http://blooge.cn/RefFinder/) [10,11,12,13,14]. This study provides a preliminary comprehensive evaluation of reference genes for *E. furcellata* under various conditions, which will be useful for subsequent gene expression and function studies.

## 2. Materials and Methods

### 2.1. Insect Rearing

*E. furcellata* was reared in the laboratory at the Institute of Entomology, Guizhou University. Male and female adults were matched 1:1 in a rearing box (15.2 cm length × 9.9 cm width × 6.3 cm height), and the eggs were placed in a small rearing box (top inner diameter 6.0 cm × bottom inner diameter 4.8 cm × and height 3.3 cm) after spawning. After the eggs hatched, they were transferred to a different rearing box (15.2 cm length × 9.9 cm width × 6.3 cm height), and reared on *Tenebrio molitor* pupae until they fledged into adults. Wet cotton balls were placed in the rearing box to maintain humidity. All rearing was carried out in an artificial climate chamber under conditions of 26 ± 0.5 °C, 60–70% relative humidity, and a photoperiod of 16/8 h = L/D.

### 2.2. Experimental Conditions

#### 2.2.1. Development Stages

Samples of *E. furcellata* at different developmental stages were collected from 1-day-old first-fifth instar nymphs and 2-day-old adult males and females. Three biological replicates were set up for each stage and each replicate, including 20 first instar nymphs, 10 second instar nymphs, 5 third instar nymphs, 3 fourth instar nymphs, 3 fifth instar nymphs, 3 female adults, and 3 male adults. The collected samples were placed in 2.0 mL Eppendorf (EP) tubes, rapidly frozen with liquid nitrogen, and stored in an ultra-low temperature refrigerator at −80 °C.

#### 2.2.2. Different Tissues

The antennae, heads (with antennae removed), thorax, abdomen, legs, and wings of 2-day-old healthy male and female adults were collected separately as different tissue samples. Three biological replicates were set up for each tissue sample, with a minimum of five insects per tissue group. The collected samples were placed in 2.0 mL EP tubes, rapidly frozen with liquid nitrogen, and stored in an ultra-low temperature refrigerator at −80 °C.

#### 2.2.3. Temperature Treatment

Three biological replicates were set up for each treatment. Five 1-day-old *E. furcellata* nymphs of the 3rd instar were collected from each replicate as sample representing different temperature treatments. The sample were stimulated for 2 h at 4 °C, 26 °C, 37 °C and 42 °C and subsequently allowed to recover at 26 °C for 30 min with *T. molitor* pupae. The collected samples were placed in 2.0 mL EP tubes, rapidly frozen with liquid nitrogen, and stored in an ultra-low temperature refrigerator at −80 °C.

#### 2.2.4. Starvation Treatment

Three biological replicates, each including 5 1-day-old *E. furcellata* nymphs of the 3rd instar, were set up for starvation treatment. The nymphs were starved for 0, 6, 12, and 24 h and collected as samples representing different starvation time treatments. The collected samples were placed in 2.0 mL EP tubes, rapidly frozen with liquid nitrogen, and stored in an ultra-low temperature refrigerator at −80 °C.

### 2.3. Total RNA Extraction and cDNA Synthesis

Total RNA was extracted from all samples using the OMEGA E.Z.N.A.^TM^ HP Total RNA Kit (Omega Bio-Tek, Norcross, GA, USA) according to the manufacturer’s instructions. The concentration and quality of the RNA were determined using a NanoPhotometerTMP-Class spectrophotometer ((Thermo Fisher Scientific, Waltham, MA, USA). The GenStar StarScript II First-strand cDNA Synthesis Mix With gDNA Remover kit (GenStar, Beijing China) was used for 1st-strand cDNA synthesis.

### 2.4. Candidate Reference Gene Selection and Primer Design

Based on the results of a homology search of these genes, nine housekeeping gene sequences—*β-1-TUB*, *RPL4*, *RPL32*, *RPS17*, *RPS25*, *SDHA*, *GAPDH2*, *EF2,* and *UBQ*—were annotated from the transcriptome data of the *E. furcellata* nymphs, and the above housekeeping genes were named according to the Blast best match terms. Primer Premier 6.0 software (PREMIER Biosoft International, Palo Alto, CA, USA) was used to design gene-specific primers, and after obtaining specific bands by PCR amplification, they were cloned, sequenced, and compared to determine the exact candidate reference gene sequences. To detect the amplification specificity, amplification efficiency, and coefficient of determination (*R*^2^) of the standard curve for each primer pair (Table 1), the cDNA obtained in Section 2.3 was used as the template to plot the melting curve for each primer pair with the standard curve of the amplification at serial concentrations using a qRT-PCR instrument.

### 2.5. qRT-PCR

All qRT-PCR reactions were performed using a CFX-96 real-time PCR system (BioRad, Hercules, CA, USA) with the GenStar 2 × RealStar Green Fast Mixture. Each 20 μL reaction consisted of 10 μL of 2 × RealStar Green Fast Mixture (GenStar), 1 μL cDNA template, 0.5 μL for forward and reverse primer (10 μmol/L), and 8 μL RNase-Free ddH_2_O. qRT-PCR was performed under the following conditions: denaturation at 95 °C for 2 min, followed by 40 cycles of dissociation at 95 °C for 15 s, annealing at 55 °C for 30 s, and extension 72 °C for 30 s. Three biological replicates and three technical replicates were set up for each sample.

### 2.6. Data Analysis

Cycle threshold (Ct) values were obtained for all biological replicates to calculate Ct averages, and the subsequent analysis was performed using the Ct averages obtained for each gene. Nine candidate reference genes were evaluated for expression stability using five methods: ∆Ct, BestKeeper, geNorm, NormFinder, and RefFinder. These different analytical methods focused on different factors. The ∆Ct method estimates the relative expression of paired genes for each sample, and the lower the mean standard deviation (SD) value, the more stably expressed the reference gene [10]. BestKeeper is used to assess the expression stability of genes by obtaining a correlation coefficient (*r*), SD, and coefficient of variation (CV) of the pairs generated between each gene. For this program, the larger the correlation coefficient, the smaller the SD and CV, and the more stably expressed the reference gene [13]. The geNorm program ranks gene expression stability by calculating the expression stability value (M) for each reference gene, judged by the criterion that the smaller the M value, the more stably expressed the reference gene. Simultaneously, the variance value (V) of the two comparisons is obtained based on the pairwise difference analysis of the standardization factor of the reference genes, and the optimal number of required reference genes is determined by the formula V*_n_*/*_n_*_+1_. When V*_n_*/*_n_*_+1_ < 0.15, the most suitable number of reference genes is *n*, and there is no need to introduce the *n*th+1st reference gene for normalization correction. However, if V*_n_*/*_n_*_+1_ > 0.15, it is necessary to add the *n*th+1st reference gene for normalization correction [11]. The computational principle of NormFinder is similar to that of geNorm. NormFinder uses an analysis of variance (ANOVA)-based model to evaluate the variation in the variation in the expression of candidate reference genes to obtain the stability value of reference gene expression. It then filters the most suitable reference genes based on the stability value, and the smaller the stability value, the more stable the gene expression [12]. Finally, the online software RefFinder was used to comprehensively evaluate the gene expression stability obtained from the above four methods and find the geometric mean to ultimately obtain a comprehensive ranking index [14].

### 2.7. Validation of Selected Reference Gene

In insects, odor-binding proteins (OBPs), which are acidic proteins, are generally found in the lymphatic fluid of the antenna sensor [15]. OBPs bind to odor molecules from the environment and transport them to odorant receptor proteins on the dendritic membrane of olfactory neurons [16]. To evaluate the effectiveness of reference gene screening, different tissues of adult male *E. furcellata* were used to verify the selected reference genes. *E. furcellata*’s *EfurOBP11* (accession no. ON505075) was used as the target gene to verify the expression stability of the reference gene (*EfurOBP11*, F: 5′-CTGTCTCCTGGCTATGGTCTT-3′, R: 5′-CTTCCCGTGTGATTTCTGCTAT-3′). According to the comprehensive ranking obtained from geNorm and RefFinder analysis, the optimal reference gene combination (*RPS17* and *RPL32*), the most stably expressed reference gene (*RPS17*), the second most stably expressed gene (*RPL32*), and the least stably expressed gene (*β-1-TUB*) were selected for standardization of the relative expression level of the target gene. qRT–PCR was performed as described above and the resulting data were analyzed using the 2^−∆∆CT^ method [17]. One-way ANOVA was used, followed by multiple comparison with Tukey’s to determine the statistical significance. Statistical differences are denoted by different letters.

## 3. Results

### 3.1. Amplification Efficiency of Primers

*E. furcellata* cDNA was used as a template after 10-fold serial dilution, and the amplification efficiency of each reference gene ranged 93.7–104.8%, with a correlation coefficient (*R*^2^) value greater than 0.990. This indicates that the primers for each candidate reference gene were reasonably designed, with good amplification efficiency and specificity. Thus, they met the requirements of fluorescence quantitative analysis and were suitable for subsequent quantitative assays (Table 1).

### 3.2. Expression Profiles of Candidate Reference Genes

**Figure 1:**
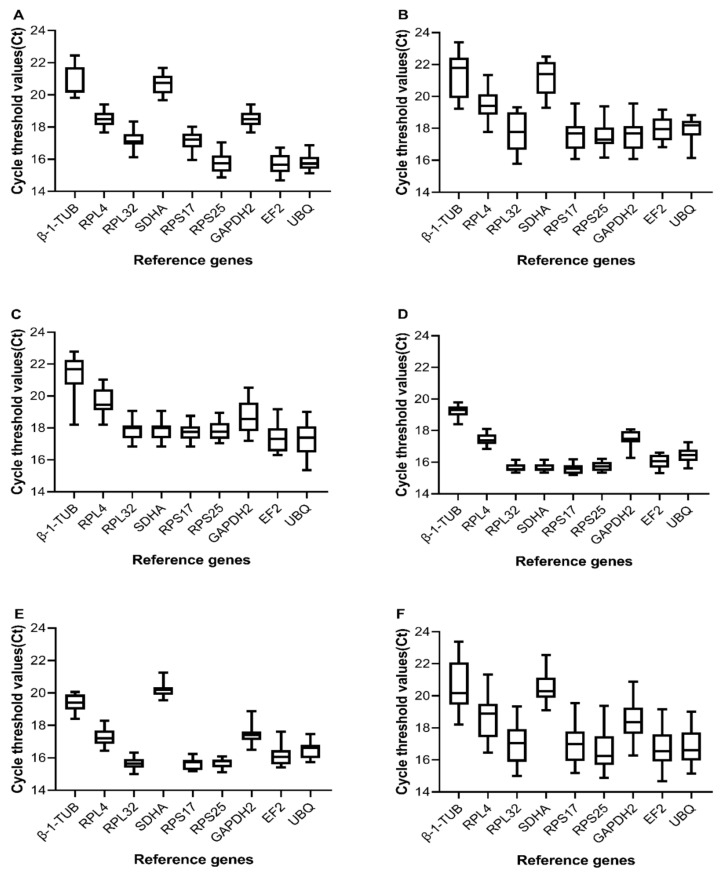
Box-and-whisker plots of the expression profiles of nine candidate reference genes in different samples of *E. furcellata* (Wolff). (**A**–**E**), the raw Ct values of the nine candidate reference genes in samples at different developmental stages (**A**) female tissues (**B**) male tissues (**C**) temperature treatments (**D**) and starvation treatments (**E**), respectively. (**F**) The Ct value distributions of the genes in all samples. Each data point represents the Ct value of each biological replicate for each treatment. The median is represented by a line in the box. The interquartile range is bordered by the upper and lower edges, which indicates the 75th and 25th percentiles, respectively. The whisker caps indicate the minimum and maximum values.

The raw Ct value is the number of cycles that the fluorescence signal of the PCR amplification product undergoes as it progresses toward a set threshold, which reflects the gene expression level. Higher Ct values indicate lower gene expression. The abundance of reference gene expression is the primary condition for screening for the reference genes. To understand the expression abundance in the *E. furcellata* samples, the Ct values of nine candidate reference genes were evaluated by qRT-PCR under five experimental conditions. As shown in Figure 1, the Ct values of all the nine genes ranged 14.68–23.39, indicating that they all exhibited high expression under different experimental conditions and met the criteria for reference gene screening. The Ct values obtained under different experimental conditions ranged as follows: 14.68–22.45, 15.79–23.39, 15.36–22.77, 15.20–20.52, and 15.00–20.06 for different developmental stages, tissues of adult females, tissues of adult males, temperature treatments, and starvation treatments, respectively. The Ct values variation of *EF2* ranged 14.68–19.17 under different experimental conditions, whereas the variation of *β-1-TUB* ranged 18.20–23.39, indicating that the expression of each candidate reference gene varied under different experimental conditions. In addition, the mean Ct values of *β-1-TUB*, *RPL4*, *RPL32*, *SDHA*, *RPS17*, *RPS25*, *GAPDH2*, *EF2*, and *UBQ* in all samples were 20.60 ± 1.36, 18.63 ± 1.23, 17.02 ± 1.17, 20.51 ± 0.88, 16.92 ± 1.08, 16.63 ± 1.15, 18.45 ± 1.06, 16.72 ± 1.12, and 16.81 ± 1.08, respectively, indicating that the mean expression level of *RPS25* was the highest and the mean expression level of *β-1-TUB* was the lowest in all samples.

### 3.3. Expression Stability of Candidate Reference Genes

To analyze the expression stability of the nine candidate reference genes in different samples, four algorithms (ΔCt, BestKeeper, geNorm, and NormFinder), were used, and the results obtained are presented in Table 2. In addition, the expression stability of candidate reference genes for the various experimental samples was ranked overall using the online tool, RefFinder.

#### 3.3.1. Developmental Stages

**Figure 2:**
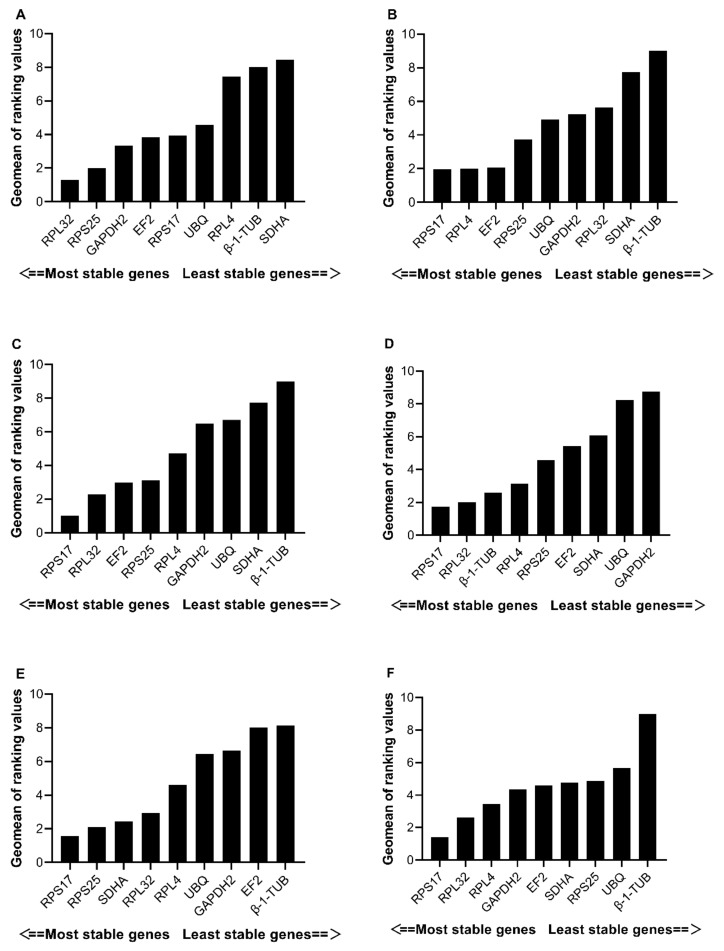
Expression stability ranking of the nine candidate reference genes under different treatment conditions as evaluated by RefFinder. (**A**–**F**) represent the results obtained from RefFinder in samples at developmental stages (**A**), in adult female tissues (**B**), in adult male tissues (**C**), under temperature treatments (**D**), under starvation treatments (**E**), and in all samples (**F**). A lower GeoMean ranking indicates more stable expression.

According to the ∆Ct analysis results, *RPL32* and *RPS25* were the most stably expressed reference genes in the samples at different developmental stages, and the NormFinder and geNorm results were consistent with those of the ∆Ct analysis (Table 2). In addition, *GAPDH2* and *UBQ* exhibited the highest expression stability when analyzed by Bestkeeper (Table 2). The RefFinder integrated stability for the reference genes at different developmental stages of *E. furcellata* was ranked as follows: *RPL32* > *RPS25* > *GAPDH2* > *EF2* > *RPS17* > *UBQ* > *RPL4* > *β-1-TUB* > *SDHA* (Figure 2). The results from different algorithms indicated that *RPL32* and *RPS25* were the most stably expressed reference genes at various developmental stages of *E. furcellata*, whereas *β-1-TUB* and *SDHA* were the least stably expressed genes.

#### 3.3.2. Female Tissues

The geNorm [11] software analysis revealed that V_2_/V_3_ value of 0.163, which is higher than 0.15; thus, the optimum number of required reference genes was 3 for different tissues of *E. furcellata* adult females. *RPL4*, *RPS17,* and *RPL32* showed the highest expression stability using geNorm; *RPS17*, *RPL4,* and *EF2* showed the highest expression stability using ∆Ct and NormFinder; and *EF2*, *UBQ,* and *RPS25* showed the highest expression stability using Bestkeeper (Table 2). The RefFinder analysis revealed an expression stability ranking of the reference genes as follows: *RPS17* > *RPL4* > *EF2* > *RPS25* > *UBQ* > *GAPDH2* > *RPL32* > *SDHA* > *β-1-TUB* (Figure 2). Since the expression of *β-1-TUB* was the most unstable in all algorithms, it could not be used as a reference gene for qRT-PCR standardization in different tissue samples of *E. furcellata* adult females.

#### 3.3.3. Male Tissues

For samples obtained from different tissues of *E. furcellata* adult males, ∆Ct and NormFinder analyses revealed *RPS17* and *EF2* as the most stably expressed genes; BestKeeper analysis revealed *RPS17* and *RPS25* as the most stably expressed genes; and geNorm revealed *RPL32* and *RPS17* as the most stably expressed reference genes. Based on the comprehensive analysis using RefFinder software, the ranking of these reference genes was as follows: *RPS17* > *RPL32* > *EF2* > *RPS25* > *RPL4* > *GAPDH2* > *UBQ* > *SDHA* > *β-1-TUB* (Figure 2). For all algorithms, the expression of *β-1-TUB* was the most unstable in different tissue samples of both *E. furcellata* adult males and females. Therefore, *β-1-TUB* is not recommended as a reference gene for qRT–PCR normalization.

#### 3.3.4. Temperature Treatments

For *E. furcellata* samples subjected to different temperature treatments, *RPS17* and *RPL32*; *RPL32* and *RPS25*; and *β-1-TUB* and *RPL4* had the most stable expression based on ∆Ct and NormFinder, BestKeeper, and geNorm analyses, respectively (Table 2). In the comprehensive ranking analysis using RefFinder, the expression stability ranking of the reference genes for different temperature conditions was as follows: *RPS17* > *RPL32* > *β-1-TUB* > *RPL4* > *RPS25* > *EF2* > *SDHA* > *UBQ* > *GAPDH2* (Figure 2). The analysis of the different algorithms revealed *RPS17* and *RPL32* as the most stably expressed reference genes in *E. furcellata* samples treated at different temperatures, whereas *UBQ* and *GAPDH2* were the least stably expressed genes.

#### 3.3.5. Starvation Treatments

In the samples subjected to different starvation time conditions, *RPS17* and *RPS25* had the highest expression stability according to ∆Ct, NormFinder, and BestKeeper analyses (Table 2), whereas *RPL32* and *SDHA* had the highest expression stability according to geNorm analysis. In the comprehensive ranking analysis using RefFinder, the stability ranking of the reference genes of *E. furcellata* at different starvation time treatments was as follows: *RPS17* > *RPS25* > *SDHA* > *RPL32* > *RPL4* > *UBQ* > *GAPDH2* > *EF2* > *β-1-TUB* (Figure 2). These results indicate that *RPS17* and *RPS25* are the most stable reference genes for analyzing samples under various starvation conditions.

#### 3.3.6. All Samples

For all samples, ∆Ct and geNorm analyses indicated that the most stably expressed reference genes were *RPS17* and *RPL32*, whereas *SDHA* and *GAPDH2* were considered the most desirable reference genes by BestKeeper analysis (Table 2). Based on NormFinder analysis, the best optimal reference genes in terms of stability were *RPS17* and *RPL4*. In addition, RefFinder analysis showed that the stability ranking of reference genes for all samples of *E. furcellata* was: *RPS17* > *RPL32* > *RPL4* > *GAPDH2* > *EF2* > *SDHA* > *RPS25* > *UBQ* > *β-1-TUB* (Figure 2). Therefore, for qRT–PCR normalization, *RPS17* and *RPL32* may be used as reference genes in all samples, whereas *β-1-TUB* cannot be used as a reference gene in any of the samples.

### 3.4. Optimal Number of Reference Genes Normalized under Different Experimental Conditions

**Figure 3:**
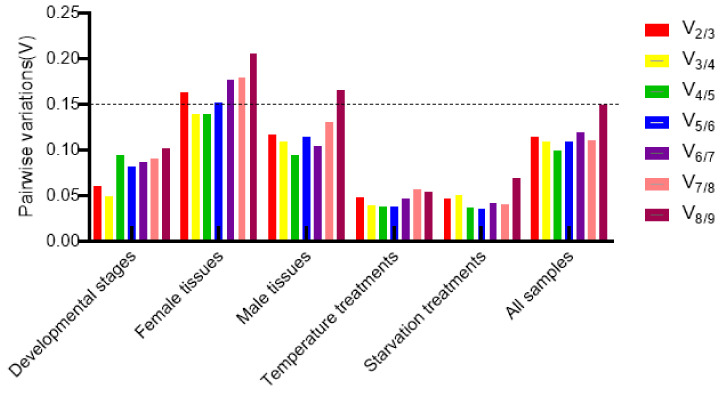
Optimal number of *E. furcellata* reference genes normalized under different experimental conditions. Pairwise variation (V*_n_*_/*n*+1_) values obtained from geNorm software were used to determine the optimal number of reference genes required for normalization of qRT–PCR data using the formula V*_n_*_/*n*+1_ < 0.15, where *n* indicates the minimum number of reference genes selected for normalization of qRT–PCR data.

To determine the minimum number of genes required to normalize qRT–PCR data, we used geNorm analysis to calculate the pairwise variance value V*_n_*/V _(*n*+ 1)_ to determine the appropriate number of reference genes. As shown in Figure 3, geNorm analysis revealed a pairwise variance value of <0.15 (V_2_/V_3_ < 0.15) in samples obtained at different developmental stages, in the tissues of adult males, and in samples subjected to various temperature and starvation treatments, indicating that two stably expressed reference genes were sufficient for standardizing qRT–PCR results. In contrast, samples from different tissues of adult females (V_2_/V_3_ > 0.15 and V_3_/V_4_ < 0.15) require three reference genes for normalization of qRT–PCR results (Figure 3). Therefore, the optimal number and combination of reference genes for different experimental conditions according to the RefFinder comprehensive reference gene ranking are as follows: *RPL32* and *RPS25* for different developmental stage samples; *RPS17*, *RPL4*, and *EF2* for different tissue samples of adult females; *RPS17* and *RPL32* for different tissue samples of adult males; *RPS17* and *RPL32* for temperature-treated 3rd instar nymph samples; *RPS17* and *SDHA* for starvation-treated 3rd instar nymph samples; and *RPS17* and *RPL32* for all samples.

### 3.5. Validation of Selected Reference Genes in E. furcellata

**Figure 4:**
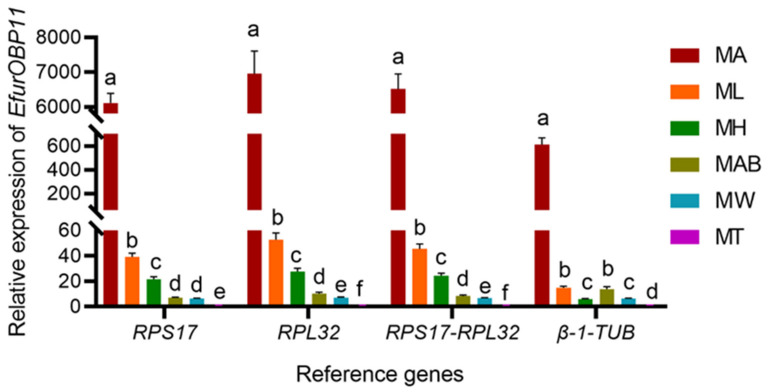
Validation of the recommended reference genes in the samples of different tissues of *E. furcellata* adult males. The relative expression level of *EfurOBP11* was normalized using the most suited (*RPS17* and *RPL32*) and the least suited (*β-1-TUB*) reference genes. MA, ML, MH, MAB, MW, and MT represent male antenna, male leg, male head, male abdomen, male wing, and male thorax, respectively. The results are depicted as the mean ± SE based on three independent biological replicates, analyzed by one-way analysis of variance (ANOVA), followed by Tukey’s multiple comparison test. The lower-case letters above the bars indicate significant differences (*p <* 0.05).

To verify the expression stability of the reference genes screened, we selected the most optimal combination of reference genes (*RPS17* and *RPL32*) for the comprehensive evaluation of their expression stability, the most stably expressed gene (*RPS17*), the second most stably expressed gene (*RPL32*), and the least stably expressed gene (*β-1-TUB*). These genes were used as reference genes to determine the relative expression levels of OBP *EfurOBP11* in different tissues of *E. furcellata* adult males. As shown in Figure 4, the target gene *EfurOBP11* showed similar expression patterns in different tissues of *E. furcellata* when standardized with *RPS17*, *RPL32*, or a combination of *RPS17* and *RPL32*. The expression level was the highest in the antennae, followed by the foot, head, abdomen, wing, and thorax. When *β-1-TUB* was used as a reference gene, although its expression level in the antennae was the highest, the relative expression level was much lower than that of the other three groups (*RPS17*, *RPL32*, and both *RPS17* and *RPL32*), and its expression level in the abdomen was higher than that of the foot and head, which was completely inconsistent with the results of the other three groups. Therefore, improper selection of reference genes in the standardization of qRT–PCR data may affect the accuracy of target gene expression levels and even lead to incorrect analysis conclusions.

## 4. Discussion

qRT-PCR is the most widely used molecular technique for gene expression analysis. It exhibits high sensitivity, high specificity, quickness, and accuracy; however, the accuracy and reliability of the results depend largely on proper data normalization using stably expressed reference genes [6,11,18]. The use of inappropriate reference genes can significantly affect quantitative results, leading to erroneous results [19,20]. In addition, several qRT–PCR studies have shown that most reference/housekeeping genes are differentially expressed under different experimental conditions and that their expression depends on the organism and the experimental conditions, suggesting that there is no “universal” reference gene [21,22,23]. Therefore, the selection of stably expressed reference genes is critical to accurate qRT-PCR analysis.

*E. furcellata* is an excellent predatory natural enemy that preys on pests, such as those belonging to the orders Lepidoptera, Coleoptera and Hymenoptera [1,2,4]. Extensive biological studies on *E. furcellata* as a natural enemy have been conducted; however, reports on reference genes for the qRT–PCR analysis of this organism are limited. Therefore, embarking on studies related to gene expression is needed to identify robust, stably expressed reference genes for *E. furcellata* under various experimental conditions to normalize the gene expression data. We screened nine putative housekeeping genes (*β-1-TUB*, *RPL4*, *RPL32*, *RPS17*, *RPS25*, *SDHA*, *GAPDH2*, *EF2*, and *UBQ*) of *E. furcellata* for expression stability under different experimental conditions. The results indicated that different housekeeping genes of *E. furcellata* are expressed at different levels under different experimental conditions, suggesting that there is no universal set of housekeeping genes that can be stably expressed toward all biotic and abiotic stresses [21,24]. This may result from the existence of specific expression profiles between different species [25,26]. Five analytical methods (ΔCt, geNorm, BestKeeper, NormFinder, and RefFinder) were used to comprehensively evaluate the expression stability of nine candidate reference genes of *E. furcellata*, which revealed not entirely consistent results. For example, based on the stability ranking of ΔCt, NormFinder, and geNorm, *RPL32* was identified as the most stably expressed reference gene at different developmental stages, whereas BestKeeper identified *GAPDH2*. This could be attributed to different calculation principles used by different analysis software [27,28]. Although the ranking order was inconsistent depending on the analysis software used, the overall trend was similar. Therefore, the overall stability ranking of candidate reference genes by the RefFinder integrated ranking software was used as a criterion, since it is a comprehensive platform that integrates four algorithms.

Ribosomal proteins are components of ribosomes and are involved in ribosome assembly and protein translation as well as play an important role in cell development [29,30]. In recent years, the ribosomal protein gene has been considered the most stably expressed reference gene under different experimental conditions and has been widely used as a reference gene for qRT–PCR analysis in insects. For example, ribosomal proteins genes exhibited stable expression in *Nilaparvata lugens* (Stål) and *Adelphocoris suturalis* [22,31]. Freitas et al. [24] demonstrated that *RPL32* and *RPS18* were the most stably expressed reference genes in three stingless bee species, including at the developmental stage, sex, and with bacterial infections and pesticide treatment. In addition, *RPL32* is also the most consistently expressed reference gene in the tissues of adult *Lygus pratensis* and in those with varying resistance to kung fu permethrin [32]. Even in *Agasicles hygrophila*, *RPL32* was confirmed to be the most stably expressed reference gene for samples obtained from different body parts [33]. In this study, four ribosomal protein genes, *RPL4*, *RPL32*, *RPS17*, and *RPS25*, were selected as candidate reference genes. A comprehensive analysis showed that the top genes consistently contained at least two ribosomal protein genes under different experimental conditions, indicating that the expression stability of ribosomal protein genes in *E. furcellata* was excellent.

The transcriptional elongation factors, another family of highly conserved proteins, play roles in binding to RNA polymerase and ensuring efficient transcription through nucleosomes [34]. In a previous study, they were selected as reference genes for normalization of qRT-PCR data, of which *EF1α* was identified as one of the most commonly used reference genes [35]. In a study by Shu et al. [26], *EF2* and *EF1α* were identified as the most stably expressed reference genes at different developmental stages of *Spodoptera frugiperda*. Bansal et al. [36] demonstrated experimentally that *EF1α* expression was stable at different developmental stages of *Aphis glycines*. In the present study, *EF2* was also stably expressed in different tissue samples of *E. furcellata* adults of both sexes.

The applicability of reference genes may vary depending on different biotic and abiotic factors (Table 2 and Figure 3). We also found that the optimal reference genes differed significantly among samples treated under different conditions, and the traditional dogma of reference genes may not always yield a stable expression effect. Therefore, it is necessary to determine the stability of reference genes under different experimental conditions before conducting *E. furcellata* gene expression studies. Prior to the development of high-throughput technologies, conventional housekeeping genes such as Tubulin and *GAPDH* were often used as reference genes and correction factors for relative quantification [37,38]. However, several experiments have demonstrated that conventionally used housekeeping genes are not necessarily stably expressed in different study subjects or in the same study subject under different experimental conditions. For example, a study by Lord et al. [39] revealed that the expression of both α-tubulin and β-tubulin was unstable after fungus-infested *Tribolium castaneum*. Furthermore, *GAPDH* was identified as the most unstable reference gene in samples of *Diabrotica virgifera* at different developmental stages [40]. As with the above findings, we observed *β-1-TUB* and *GAPDH2* were also the most unstable housekeeping genes although *GAPDH2* was more stable at different developmental stages and *β-1-TUB* was more stable at different temperatures, compared with other genes in this article, respectively. Our results indicate that *β-1-TUB* and *GAPDH2* cannot be recommended as reference genes for *E. furcellata* qRT–PCR experiments.

In many previous studies, only one reference gene was generally selected to normalize qRT–PCR data results. However, owing to increased in-depth studies on the expression stability of reference genes, researchers have begun to normalize qRT–PCR results with two or more reference genes as a single reference gene may not be sufficient [41]. In addition, the overuse of reference genes can reduce the accuracy of standardized qRT-PCR results [42]. Therefore, it is necessary to evaluate the optimal number of reference genes under different conditions. In this study, the optimal number of reference genes was determined by the number of paired variants using the geNorm program (Figure 3). The V_2_/V_3_ was less than 0.15 at different developmental stages, in the tissues of adult males, and under different temperature and starvation treatments, indicating the requirement of only two reference genes for qRT–PCR result normalization; conversely, the V_2_/V_3_ was more than 0.15 in the tissues of adult females, wherein three reference genes were needed. We also found that among the stable values obtained from the calculation of the expression of nine candidate reference genes by different algorithms, the stable values were relatively larger for samples obtained from different tissues of adult females, indicating that the female sex had a greater influence on the stable expression of reference genes. The obtained results also reflected that it is crucial to introduce appropriate reference genes for correcting the accuracy of gene expression under different conditions; however, the use of reference genes itself may introduce more instability. Thus, too many reference genes should not be introduced [42]. In particular, after the addition of the fourth reference gene, the gene expression stability decreases and the correction accuracy and overall accuracy is reduced. Therefore, it is proposed that the number of optimal reference genes in a combination should not exceed 3 [43]. When screening for optimal reference gene combinations, we usually use up to three stably expressed reference genes as a combination for the calibration of fluorescence quantitation. Combined with RefFinder, the expression stability of each reference gene predicted by different analysis methods is comprehensively sorted, and the optimal selection of the reference gene may be finally determined. This avoids the biasedness of single software analysis and results in more reliable screening results. Therefore, the optimal gene combination for different tissues of *E. furcellata* adult females is *RPS17*, *RPL4*, and *EF2*. The optimal reference gene combination for different developmental stages, in the tissues of adult males, under temperature treatments, under starvation treatment, and for all samples was *RPL32* + *RPS25*, *RPS17* + *RPL32*, *RPS17* + *RPL32*, *RPS17* + *RPS25*, and *RPS17* + *RPL32*, respectively. Thus, it is evident that the expression of ribosomal protein genes in *E. furcellata* is highly stable under different experimental conditions.

## 5. Conclusions

*E. furcellata* is an excellent natural predatory enemy. It plays an important role in the development of agricultural security and represents a green environmental solution. Therefore, gene expression analyses and functional genomics of *E. furcellata* will further enhance its applicability. This study is the first to screen reference genes of *E. furcellata*, which is of great significance for future studies on the expression of its related genes and to ensure the reliability of the gene expression data. The expression stability of nine candidate reference genes under biotic (developmental stage and tissue) and abiotic (temperature and starvation stress) conditions was determined using five commonly used algorithms (ΔCt, BestKeeper, NormFinder, geNorm, and RefFinder). Our results show that no universal reference gene was stably expressed under all experimental conditions, rather a combination of the most stable reference genes was optimal. These included *RPL32* and *RPS25* at different developmental stages; *RPS17*, *RPL4*, and *EF2* for adult female tissues; *RPS17* and *RPL32* for adult male tissues; *RPS17* and *RPL32* for different temperature treatments of nymphs; *RPS17* and *RPS25* for nymph samples under starvation stress; and *RPS17* and *RPL32* for all samples. This study provides the most basic and important steps for standardizing qRT–PCR analysis in *E. furcellata*, lays a foundation for further molecular biology studies, and provides a reference for the rational selection of qRT–PCR reference genes.

## Figures and Tables

**Table 1:** Details of the primer pairs used for qPCR in *Eocanthecona furcellata* (Wolff).

Gene	Accession Number	Primer Sequence (5′–3′)	Product Length (bp)	Tm (°C)	Efficiency (%)	*R* ^2^
*β-1-TUB*	ON505066	F: ACTGACACATTCTCTTGGAGGT R: GGTGGCGTTATAAGGTTCTACA	157	55.0	101.1	0.996
*RPL4*	ON505068	F: TTCCCGAAATCCCTCTTGTTG R: GTCTCCTATTACGCATCTTACCT	162	55.0	97.5	0.996
*RPL32*	ON505067	F: AGGAGGAACTGGCGTAAGC R: GGAACTAACAGCATGAGCGATT	213	55.0	103.3	0.996
*SDHA*	ON505070	F: GCTCCAGAACTTAATGTTGTGT R: TACGCCAATGCTCCTCAATAG	170	55.0	102.6	0.995
*RPS17*	ON505073	F: CGCTATCATTCCTACCAAACCT R: CTCCAACATCTTCAACATTCCA	217	55.0	95.0	0.997
*RPS25*	ON505074	F: GTCTCCTATTACGCATCTTACCT R: CTGCTTTAGTCGCCCTGGTA	115	55.0	93.7	0.995
*GAPDH2*	ON505069	F: TCTGTGGTGTCAACTTGGATG R: CGTCTTCTGAGTAGCGGTAAC	167	55.0	99.7	0.996
*EF2*	ON505072	F: TGGAGGTATCTATGGTGTACTGA R: AATGGCTTGGTGTTGGTGTC	228	55.0	104.8	0.995
*UBQ*	ON505071	F: CGGCAAGACTATCACACTAGAAG R: ATACCTCCTCTGAGACGAAGTAC	204	55.0	100.5	0.990

Note: Abbreviation: *β-1-TUB*, tubulin beta-1; *RPL4*, 60S ribosomal protein L4; *RPL32*, 60S ribosomal protein L32; *SDHA*, succinate dehydrogenase; *RPS17*, 40S ribosomal protein S17; *RPS25*, 40S ribosomal protein S25; *GAPDH2*, glyceraldehyde-3-phosphate dehydrogenase 2; *EF2*, elongation factor 2, and *UBQ*, ubiquitin. The abbreviations are exactly the same as Table 2 and Figures 1, 2 and 4.

**Table 2:** Expression stability of the candidate reference genes under different experimental conditions.

Experimental Conditions	Reference Gene	∆Ct		BestKeeper		NormFinder		geNorm	
		Stability	Rank	Stability	Rank	Stability	Rank	Stability	Rank
Developmental stages	*β-1-TUB*	0.904	8	0.789	8	0.785	8	0.622	8
	*RPL4*	0.802	7	0.888	9	0.662	7	0.549	7
	*RPL32*	0.542	1	0.422	3	0.153	1	0.306	1
	*SDHA*	0.992	9	0.544	7	0.901	9	0.705	9
	*RPS17*	0.574	4	0.48	5	0.248	3	0.332	4
	*RPS25*	0.556	2	0.477	4	0.217	2	0.306	1
	*GAPDH2*	0.674	5	0.377	1	0.417	5	0.427	5
	*EF2*	0.567	3	0.525	6	0.256	4	0.313	3
	*UBQ*	0.728	6	0.388	2	0.53	6	0.486	6
Female tissues	*β-1-TUB*	1.915	9	1.207	9	1.774	9	1.267	9
	*RPL4*	1.055	2	0.739	4	0.614	2	0.434	1
	*RPL32*	1.211	6	1.04	8	0.933	7	0.543	3
	*SDHA*	1.497	8	0.961	7	1.176	8	1.082	8
	*RPS17*	1.03	1	0.788	5	0.633	3	0.434	1
	*RPS25*	1.109	4	0.704	3	0.713	4	0.618	4
	*GAPDH2*	1.174	5	0.931	6	0.780	5	0.698	5
	*EF2*	1.102	3	0.631	1	0.552	1	0.791	6
	*UBQ*	1.309	7	0.647	2	0.906	6	0.944	7
Male tissues	*β-1-TUB*	1.525	9	1.03	9	1.413	9	0.966	9
	*RPL4*	0.924	5	0.613	4	0.657	5	0.529	5
	*RPL32*	0.796	3	0.471	3	0.468	3	0.264	1
	*SDHA*	1.146	8	0.825	7	0.905	8	0.806	8
	*RPS17*	0.737	1	0.423	1	0.326	1	0.264	1
	*RPS25*	0.824	4	0.444	2	0.508	4	0.358	3
	*GAPDH2*	0.976	7	0.813	6	0.714	7	0.623	6
	*EF2*	0.794	2	0.671	5	0.361	2	0.468	4
	*UBQ*	0.97	6	0.846	8	0.661	6	0.693	7
Temperature treatments	*β-1-TUB*	0.407	3	0.302	5	0.247	3	0.24	1
	*RPL4*	0.418	4	0.316	6	0.276	4	0.24	1
	*RPL32*	0.398	2	0.21	1	0.22	2	0.285	4
	*SDHA*	0.484	7	0.288	4	0.359	7	0.384	7
	*RPS17*	0.376	1	0.26	3	0.173	1	0.253	3
	*RPS25*	0.477	6	0.241	2	0.349	6	0.351	6
	*GAPDH2*	0.598	9	0.374	8	0.517	9	0.461	9
	*EF2*	0.460	5	0.327	7	0.336	5	0.316	5
	*UBQ*	0.529	8	0.410	9	0.42	8	0.422	8
Starvation treatments	*β-1-TUB*	0.653	9	0.407	6	0.575	9	0.509	9
	*RPL4*	0.465	4	0.416	7	0.286	4	0.351	4
	*RPL32*	0.479	5	0.292	3	0.313	5	0.301	1
	*SDHA*	0.458	3	0.365	4	0.282	3	0.301	1
	*RPS17*	0.404	1	0.284	2	0.114	1	0.333	3
	*RPS25*	0.437	2	0.244	1	0.221	2	0.375	5
	*GAPDH2*	0.516	6	0.439	9	0.381	6	0.399	6
	*EF2*	0.636	8	0.426	8	0.553	8	0.468	8
	*UBQ*	0.535	7	0.406	5	0.409	7	0.425	7
All samples	*β-1-TUB*	1.374	9	1.182	9	1.180	9	1.050	9
	*RPL4*	0.946	3	1.079	8	0.604	2	0.510	3
	*RPL32*	0.942	2	0.973	6	0.622	4	0.355	1
	*SDHA*	1.266	8	0.729	1	1.042	8	0.958	8
	*RPS17*	0.879	1	0.913	4	0.488	1	0.355	1
	*RPS25*	0.946	4	1.002	7	0.626	5	0.631	4
	*GAPDH2*	0.962	6	0.859	2	0.628	6	0.707	5
	*EF2*	0.972	5	0.913	4	0.621	3	0.770	6
	*UBQ*	1.127	7	0.909	3	0.860	7	0.857	7

## Data Availability

The data and materials supporting the conclusions of this study are included within the article.
